# Mycotoxins Co-Exposure Risk Assessment in Coix Seed: Contamination Levels and Safety for Dietary Consumption and Medicinal Intake

**DOI:** 10.3390/foods14223965

**Published:** 2025-11-19

**Authors:** Yue Han, Lulu Wang, Qingsong Yuan, Lanping Guo, Chuanzhi Kang, Ye Yang, Chenghong Xiao, Changgui Yang, Jinqiang Zhang, Tao Zhou

**Affiliations:** 1 Resource Institute for Chinese and Ethnic Materia Medica, Guizhou University of Traditional Chinese Medicine, Guiyang 550025, China; hanyuejxy@163.com (Y.H.); 15885631997@163.com (L.W.); yqs198609031006@126.com (Q.Y.); xiaochenghong1986@126.com (C.X.); 15285135045@139.com (C.Y.); 2 Guizhou Key Laboratory for Germplasm Innovation and Resource-Efficient Utilization of Dao-Di Herbs, Guiyang 550025, China; 3 State Key Laboratory for Quality Ensurance and Sustainable Use of Dao-Di Herbs, Beijing 100700, China; glp01@126.com (L.G.); kangchuanzhi1103@163.com (C.K.); 4 Faculty of Life Science and Technology, Kunming University of Science and Technology, Kunming 650500, China; yangyekm@163.com

**Keywords:** coix seed, mycotoxins, health risk, dietary consumption, multi-mycotoxins co-exposure risk, medicinal intake

## Abstract

Coix seed, a traditional medicinal and edible crop, is susceptible to mycotoxin contamination posing potential health risks, yet systematic risk assessments for its dual dietary and medicinal pathways remain limited. Fifty batches were collected from five major production regions in China. UPLC-MS/MS was used to quantify eight mycotoxins in raw materials and decoctions. A Monte Carlo simulation model assessed long-term (20-year) health risks via both pathways, and acceptable daily intake (ADI) levels were derived using a combined margin of exposure (MOE) threshold of 10,000. Results indicated that mycotoxins were present in 94% of samples. Zearalenone (ZEN) was the most frequent, with an occurrence of 82% and concentrations of 52.16~1804.43 μg/kg. Dietary exposure indicated potential risks for ZEN (MOE = 259), aflatoxin B1 (AFB1, MOE = 666), and AFB2 (MOE = 8040). Six mycotoxins transferred to decoctions at rates of 0.70~19.73%, with long-term medicinal use indicating potential ZEN-related risk (MOE = 4880). Multi-mycotoxin co-exposure assessment revealed elevated dietary risk (combined MOE = 181), whereas medicinal exposure within a standard 3-month treatment course remained within acceptable limits. Safety intake thresholds are proposed: ≤30 g/day for ≤130 days (dietary) and ≤30 g/day for ≤2000 days (medicinal). This study establishes a risk assessment framework applicable to herbal materials with both dietary and medicinal applications.

## 1. Introduction

The Coix seed refers to the dried mature seed of *Coix lacryma-jobi* L. var. ma-yuen (Roman.) Stapf, a member of the Poaceae family. As a traditional food and medicinal resource, the Coix seed contains various nutrients, including fatty acids, proteins, vitamin E, and amino acids [1]. It can be consumed in the form of porridge, soup, or pastries, serving as a source of essential energy and nutrients for the human body [2,3]. Coix seed also possesses significant pharmacological properties. It contains bioactive compounds, such as polysaccharides and flavonoids, which are responsible for its anti-tumor activity, hypoglycemic effects, modulation of intestinal microbiota, and anti-inflammatory actions. It is widely used in traditional Chinese medicine to treat conditions including edema, lung abscess, and intestinal abscess, highlighting its substantial clinical value [4,5,6]. In recent years, Coix seed has attracted considerable attention across various domains, including health foods, medicinal cuisine [7].

However, like other cereal crops, Coix seed is susceptible to contamination by various microorganisms, including *Aspergillus* and *Fusarium* species, during cultivation, processing, and storage. Mycotoxins, as secondary metabolites produced by these microorganisms, pose a potential threat to the safety of Coix seed. These toxins primarily include aflatoxins (AFB1, AFB2), zearalenone (ZEN), deoxynivalenol (DON), sterigmatocystin (ST), T-2/HT-2 toxins and ochratoxin A (OTA) [8,9]. AFB1 has been classified by the International Agency for Research on Cancer (IARC) as a Group 1 carcinogen, which is capable of inducing hepatocellular carcinoma through the mechanism of DNA adduct formation [10]. ZEN, functioning as an estrogen analog, can disrupt the endocrine system in animals and humans, leading to reproductive toxicity [11]. DON and T-2/HT-2 toxins primarily cause gastrointestinal inflammation and hematopoietic system damage [12,13]. OTA exhibits nephrotoxicity and immunosuppressive effects, with long-term exposure being closely associated with Balkan endemic nephropathy [14]. It is noteworthy that Coix seeds are susceptible to simultaneous contamination by multiple mycotoxins, which may potentially exhibit synergistic effects [15]. From a food safety perspective, co-exposure of mycotoxins increases toxicological hazards [16]. However, current research lacks comprehensive risk assessment of mycotoxin co-exposure in Coix seed, potentially leading to an underestimation of its health risks.

In addition, current health risk assessments of coix seeds primarily focus on their consumption, while risk assessments for the medicinal use of Coix seed decoctions have not yet been conducted [17,18,19]. In fact, there are significant differences in the preparation methods, dosages, and applicable populations when Coix seed is used in food and medicine. Therefore, the health risks brought by dietary consumption cannot represent the risks when it is used as medicine [20]. In medicinal applications, Coix seed is typically decocted, and the resulting aqueous extract is consumed, with health risks primarily determined by the transfer rate of mycotoxins from the raw material to the decoction [21]. Therefore, it is imperative to conduct separate health risk assessments specifically for the medicinal application of Coix seed to mitigate potential health risks associated with mycotoxins contamination in this vulnerable consumer group.

To address this critical knowledge gap, the present investigation implemented a dual analytical paradigm. For dietary risk evaluation, the QuEChERS (Quick, Easy, Cheap, Effective, Rugged, and Safe) procedure using organic solvents was systematically applied to ensure comprehensive mycotoxin extraction from raw Coix seeds, thereby capturing maximum contamination levels for conservative exposure assessment. Owing to its operational simplicity, high efficiency, and excellent recovery rates, QuEChERS-based methodologies have been increasingly adopted in the analysis of multiple mycotoxins in herbal medicine matrices, demonstrating strong applicability for complex sample systems [22]. Concurrently, medicinal risk assessment involved aqueous decoction preparation under pharmacopeia-specified conditions to simulate clinical usage patterns and quantify actual mycotoxin exposure in therapeutic applications.

Building upon these methodological foundations, this study established three principal objectives: (1) quantitative determination of major mycotoxins in both raw Coix seeds and their aqueous extracts; (2) comprehensive health risk assessment across dietary and therapeutic exposure routes; and (3) derivation of scenario-specific acceptable daily intake (ADI) values to inform regulatory frameworks for this dual-use commodity. These investigations aim to create an evidence-based safety profile addressing both nutritional and pharmacological utilization of Coix seeds.

## 2. Materials and Methods

### 2.1. Chemical and Standards

Pre-treatment reagents included sodium chloride (Lot No.: 20200919) from Shanghai Hushi Co., Ltd. (Shanghai, China); anhydrous magnesium sulfate (Lot No.: 22030) from Tianjin Zhiyuan Chemical Reagent Co., Ltd. (Tianjin, China); octadecylsilyl-bonded silica gel (ODS, Lot No.: 17606) and N-propyl ethylenediamine (PSA, Lot No.: P20-00557, particle size distribution is 40–60 µm and the pore size is 60 Å.) from Tianjin Comio Chemical Reagent Co., Ltd. (Tianjin, China); and graphitized carbon black (GCB, Lot No.: C11495332) from Shanghai Macklin Biochemical Technology Co., Ltd. (Shanghai, China). Chromatographic grade methanol (Lot No.: 200343), acetonitrile (Lot No.: 202888), and formic acid (Lot No.: 166511) were obtained from Fisher Scientific (Waltham, MA, USA).

The mycotoxin reference standards, including Aflatoxin B1 (AFB1, Lot No.: MSS1003), Aflatoxin B2 (AFB2, Lot No.: MSS1014), Aflatoxin G1 (AFG1, Lot No.: MSS1005), Aflatoxin G2 (AFG2, Lot No.: MSS1006), Ochratoxin A (OTA, Lot No.: MSS1020), Sterigmatocystin (ST, Lot No.: MSS1022), Zearalenone (ZEN, Lot No.: MSS1024), and T-2 Toxin (T-2, Lot No.: MSS1023), were all procured from Qingdao Pribolab Co., Ltd. (Qingdao, China). All compounds exhibited a purity greater than 99%.

### 2.2. Sample Collection

Coix seed samples were procured from five traditional Chinese medicine markets in China. All samples were transported to the laboratory in sterile, airtight bags and stored at 4 °C inhibit mycotoxin production and fungal growth until pretreatment [23,24].

### 2.3. QuEChERS Sample Preparation

Based on prior research [25,26,27], the QuEChERS pretreatment method was systematically optimized by evaluating key parameters, including extraction solvent, extraction method, extraction duration, and purifying agent (Figure S1A–D). 2.0 g of Coix seed powder was weighed and mixed with 20.0 mL extraction solvent (acetonitrile:water:formic acid, 80:19:1, *v*/*v*/*v*). The mixture was vortexed and extracted with shaking (180 r·min^−1^) for 1 h. Subsequently, 2 g of MgSO_4_ and 1 g of NaCl were added, followed by vortexing for 1 min. The solution was centrifuged at 5000 r·min^−1^ for 5 min. A 5 mL aliquot of the supernatant was transferred and mixed with purification materials (0.3 g C18 + 0.1 g PSA + 0.3 g MgSO_4_ + 0.01 g GCB), then vortexed for 1 min. A 3 mL portion of the purified supernatant was collected, concentrated to near dryness under nitrogen at 40 °C, and reconstituted with 1.0 mL of 50% acetonitrile. The final solution was filtered through a 0.22 µm membrane filter prior to analysis and stored at 4 °C for no more than 24 h before LC–MS/MS injection.

### 2.4. Decoction Sample Preparation

The preparation of the Coix seed decoction was conducted by simulating the traditional decoction method of Chinese herbal medicine. Precisely 30 g of Coix seed sample was weighed and decocted with 200 mL of water for 20 min, followed by filtration [28]. An additional 200 mL of water was added for a second 20 min decoction, and the two decoctions were combined. The final volume was adjusted to 500 mL with distilled water. The final test sample was prepared by thorough mixing of the decoction, followed by storage at 4 °C and processing within 48 h. Subsequently, a 10 mL aliquot was aseptically withdrawn from the center of the container, transferred into a sterile 50 mL centrifuge tube, and centrifuged at 4000 rpm for 10 min. Following centrifugation, 5 mL of the supernatant was collected and prepared into the test solution according to the procedures detailed in Section 2.5.

### 2.5. Mycotoxins Determination by HPLC-MS/MS

The ultra-performance liquid chromatography-tandem mass spectrometry system (SCIEX-QTRAP 5500, SCIEX, Framingham, MA, USA), electronic analytical balance (EL104, Mettler Toledo, Greifensee, Switzerland), benchtop refrigerated centrifuge (Centrifuge 5810R, Eppendorf, Germany), homogenizer (PRB-2000S, Qingdao Pribolab Co., Ltd., Qingdao, China), and shaking incubator (BSD-150, Shanghai Boxun Medical Biological Instrument, Shanghai, China) were used.

AFB1, AFB2, OTA, and ST were prepared as single standard stock solutions of 1.0 mg·mL^−1^ using 50% acetonitrile; HT-2, T-2, DON, and ZEN were prepared as single standard stock solutions of 1.25 mg·mL^−1^ using 50% acetonitrile. Precisely 0.1 mL of the above single standard stock solutions was transferred to a 10.0 mL volumetric flask to obtain ZEN single standard stock solution at 12.5 µg·mL^−1^ and other toxin single standard stock solutions at 10 µg·mL^−1^. Then, 1.0 mL of each single standard stock solution was transferred to a 25 mL volumetric flask to prepare a mixed reference solution containing 400 ng·mL^−1^ of AFB1, AFB2, ST, OTA and 500 ng·mL^−1^ of HT-2, T-2, DON, ZEN. The differing concentrations were used to account for the variations in mycotoxin potency, regulatory limits, and expected contamination levels, thereby ensuring optimal analytical response and accurate quantification for all target analytes within their relevant ranges.

The chromatographic analysis was performed using a Waters HSS T3 column (100 × 2.1 mm, 1.8 µm) at a column temperature of 40 °C. The mobile phase consisted of water containing 0.1% formic acid and acetonitrile, with gradient elution applied. The flow rate was set at 0.3 mL·min^−1^, and the injection volume was 5 µL. The mass spectrometry conditions employed an electrospray ionization (ESI) source in positive ion mode, with a capillary voltage of 3.5 kV, ion source temperature of 150 °C, cone gas flow of 30 L·h^−1^, desolvation temperature of 500 °C, and desolvation gas flow of 600 L·h^−1^. Data acquisition was performed in multiple reaction monitoring (MRM) mode, and the mass spectrometry parameters for each mycotoxin reference solution are listed in Table S1.

### 2.6. Method Validation

Prior to the analysis of raw Coix seed materials and their aqueous decoctions, methodological validation was conducted in accordance with the requirements stipulated in the “Guidelines for the Validation of Analytical Methods for Sample Determination” (General Chapter 9101, Chinese Pharmacopoeia, 2020 Edition).

For specificity, eight mycotoxins (AFB1, AFB1, OTA, ZEN, DON, HT-2, T-2, ST) exhibited well-resolved chromatographic peaks with no interference from matrix impurities, enabling accurate identification of target analytes (Figure S2). Regarding linearity and matrix effects, Raw Coix seed exhibited a matrix effect of 67.78~120.87% for the tested toxins, while Coix seed decoction showed a matrix effect of 73.52~127.13% (the conventional normal range of matrix effect is 70~120%); the two matrices caused interference of varying degrees in mycotoxin detection. To mitigate such interference and achieve accurate quantification, matrix-matched calibration curves were adopted, with correlation coefficients (R^2^) ranging from 0.9949 to 0.9994 for raw Coix seed and from 0.9974 to 0.9996 for Coix seed decoction, indicating excellent linearity (Tables S2 and S3). With respect to sensitivity, the limit of detection (LOD) was 0.010~0.611 μg·kg^−1^ for raw Coix seed and 0.009~1.163 μg·kg^−1^ for Coix seed decoction, while the limit of quantitation (LOQ) was 0.021~1.354 μg·kg^−1^ and 0.020~2.532 μg·kg^−1^, respectively, satisfying the required sensitivity for trace analysis (Tables S2 and S3). For precision and stability, relative standard deviations (RSDs) for repeatability, intermediate precision, and stability across both sample matrices were below 6%, indicating high methodological consistency (Table S4). In the spiked recovery experiments (*n* = 3) at three fortification levels, mean recoveries of the 8 mycotoxins differed between the two matrice. For raw Coix seed, the recoveries ranged from 70.88% to 116.20 with RSDs of 1.5~3.6%; for Coix seed decoction, the recoveries were in the range of 87.3% to 113.4% with RSDs of 0.95~3.12%. All RSDs were <15%, confirming acceptable accuracy for both raw Coix seed and its decoction (Table S5).

### 2.7. Risk Assessment

In accordance with the health risk assessment model for chemical contaminants issued by the United States Environmental Protection Agency (USEPA), an assessment model was developed using the Monte Carlo simulation method and probabilistic assessment approach [29]. This model is employed to simulate the exposure levels of residents consuming Coix seed to major mycotoxin contaminants, expressed as the estimated daily intake (EDI). Various exposure durations (ED) and the transfer rate (T) of mycotoxins from raw Coix seed to its decoction are incorporated into the assessment, as detailed in Equations (1) and (2) [21].

(1) $$EDI=\frac{C\times IC\times T}{BW}\times \frac{ED}{AY}$$

(2) $$T=\frac{MD}{MR}\times 100\%$$

where EDI denotes the estimated daily intake (μg/kg bw/d); C represents the average mycotoxin content in Coix seed decoction (μg/kg); IC refers to the average daily consumption of Coix seed (kg/d). Statistical data from 2018 to 2021 show that IC is approximately 0.03 kg/d [18]. BW denotes average body weight (kg), which is reported as 63 kg according to the China Pharmacopoeia Commission [28]; ED refers to the exposure duration; and AY represents the average life expectancy (years), set at 70 years [21]. T indicates the transfer rate from raw material to decoction (%), where MR represents the mycotoxin content in the raw material and MD represents the mycotoxin content in the decoction. In assessments involving raw materials rather than decoctions, the T value is assigned as 1.

According to the report of the European Food Safety Authority (EFSA), the margin of exposure (MOE) approach is used for risk characterization [10]. In this study, to systematically assess the risk associated with co-exposure by multiple mycotoxins, the MOE method was applied to evaluate the risk of all included mycotoxins. A lower MOE value indicates greater health risk. Specifically, an MOE exceeding 10,000 is generally considered to represent an acceptable risk level; an MOE between 100 and 10,000 suggests a potential concern; and an MOE below 100 indicates a high level of risk [30].

MOE is defined as the ratio of the benchmark dose lower confidence limit (BMDL_10_) or the no-observed-adverse-effect level (NOAEL) to the estimated daily intake (EDI), as calculated using Equation (3). The risk assessment of mycotoxins co-exposure was performed by determining the combined MOE value (MOEt/MOET), in accordance with EFSA guidelines [31], using Equations (4) and (5).

(3) $$MOE=\frac{{BMDL}_{10}}{EDI}\;\mathrm{or}\;\frac{NOAEL}{EDI}$$

(4) $${MOE}_{t}={\left(\frac{1}{{MOE}_{1}}+\frac{1}{{MOE}_{2}}+\cdots +\frac{1}{{MOE}_{n}}\right)}^{-1}$$

(5) $${MOE}_{T}={\left(\frac{1}{{MOE}_{t1}}+\frac{1}{{MOE}_{t2}}+\cdots +\frac{1}{{MOE}_{tn}}\right)}^{-1}$$

where BMDL_10_ denotes the lower 95% confidence limit of the benchmark dose associated with a 10% increase in cancer or non-cancer risk; NOAEL refers to the no-observed-adverse-effect level; MOEt represents the exposure threshold for co-exposure of *n* mycotoxins, calculated based on the average contamination levels of each mycotoxin across the entire batch of samples; and MOET indicates the exposure threshold for the overall sample set, determined by the characteristics of different mycotoxin contamination combinations. Based on the reports from EFSA [10,32,33,34], the BMDL_10_ and NOAEL values for these toxins were collected as Table S6.

### 2.8. Acceptable Daily Intake (ADI) Estimation

Based on the modeling framework for combined pollution exposure risk (Equations (1)–(5)), a threshold value of MOEt/MOET equal to 10,000 was established. Subsequently, the intake limits of Coix seed for both dietary and medicinal intake were inversely calculated across various time periods—1 month, 3 months, 6 months, 1 year, 5 years, 10 years, 20 years, 40 years, and 70 years—to ensure that consumer exposure to these mycotoxins through Coix seed consumption at this contamination level does not pose health risks.

### 2.9. Statistical Analysis

Based on the Monte Carlo simulation method, Oracle Crystal Ball assessment software (Version 4.0.2) was employed to evaluate the concentration of each toxin in the measured samples. The Anderson–Darling test and Kolmogorov–Smirnov (K-S) test were performed to identify the best-fit distribution model, which was subsequently used to calculate the exposure levels of mycotoxins in Coix seed. For samples in which mycotoxins were not detected (i.e., below the limit of detection (LOD)), a concentration of zero was assigned. For samples with results falling between the LOD and the limit of quantification (LOQ), a value of half the LOQ was used in the calculations. The number of iterations was set to 10,000, and 200 bootstrap samples were generated by default. From the simulation results, the 50th percentile (median), mean, and 95th percentile values of EDI and MOE were extracted for further analysis. Generally, a duration of 20 years is considered representative of long-term exposure [21]. Therefore, the EDI and MOE values derived from the 20-year risk assessment data were selected as the default parameters for subsequent analysis. GraphPad Prism 8.0 and Origin 2021 software were utilized for statistical analysis and data visualization in this study.

## 3. Results

### 3.1. Risk Assessment for ZEN in Coix Seed for Dietary Consumption and Medicinal Intake

**Figure 1:**
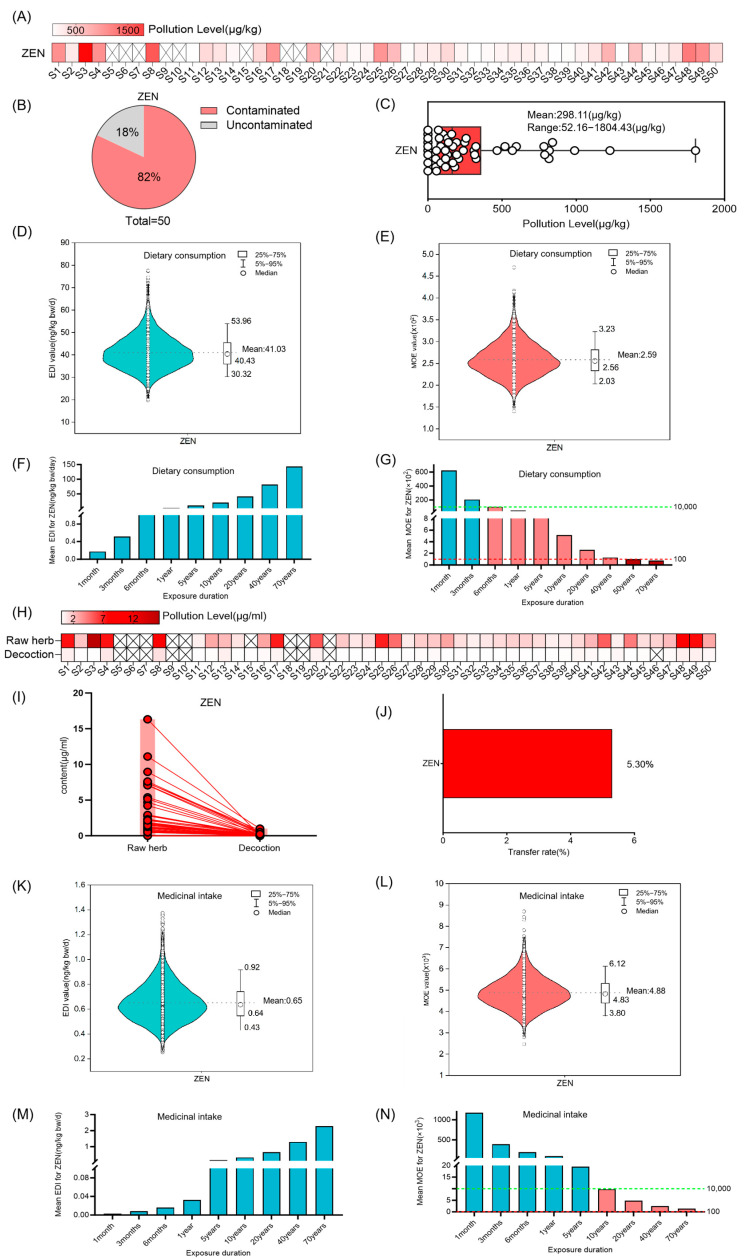
Risk assessment for ZEN in Coix seed for dietary consumption and medicinal intake. (**A**–**C**) Concentration and occurrence of ZEN in 50 random Coix seed samples. The symbol “×” denotes concentrations below the limit of quantification (LOQ). (**D**,**E**) The distribution of Estimated Daily Intake (EDI) and Margin of Exposure (MOE) of ZEN in Coix seed via dietary exposure for 20 years (10,000 simulations). The violin shape represents the density distribution of EDI/MOE values. The dashed line marks the mean value as a reference. (**F**,**G**) The average EDI and MOE of ZEN in Coix seed for dietary consumption over different exposure durations. The dashed green line marks the MOE reference value of 10,000, and the dashed red line marks the MOE reference value of 100. Different colors of bars represent distinct MOE ranges: teal bars indicate MOE > 10,000, pink bars indicate MOE between 100 and 10,000, and red bars indicate MOE < 100. (**H**–**J**) Quantification of ZEN in 50 random Coix seed samples and their decoctions, and analysis of its transfer (concentration and rate) from the raw herb to the decoction. The symbol “×” denotes concentrations below the limit of quantification (LOQ). Each circle represents the mycotoxin detection value of an individual sample, and the lines connect the paired samples from the same batch. (**K**,**L**) The distribution of Estimated Daily Intake (EDI) and Margin of Exposure (MOE) of ZEN in decoction via medicinal exposure for 20 years (10,000 simulations). The violin shape represents the density distribution of EDI/MOE values. The dashed line marks the mean value as a reference. (**M**,**N**) The average EDI and MOE of ZEN in the decoction of Coix seed for medicinal intake over different exposure durations. The dashed green line marks the MOE reference value of 10,000, and the dashed red line marks the MOE reference value of 100. Different colors of bars represent distinct MOE ranges: teal bars indicate MOE > 10,000, pink bars indicate MOE between 100 and 10,000.

Among the 50 random samples of Coix seed, ZEN was quantified in 41 (82.0%), with concentrations ranging from 52.16 to 1804.43 µg/kg and an average level of 298.1 ± 369.9 µg/kg (Figure 1A–C). The risk assessment results showed that the average daily intake of ZEN gradually increased with exposure time. When exposed through dietary consumption for 20 years, the average EDI was 41.03 ng/kg bw (50th = 30.32 ng/kg bw, 95th = 53.96 ng/kg bw) (Figure 1D,F). The MOE value exhibited a progressive decline corresponding to increased exposure duration. Under prolonged dietary exposure exceeding 6 months, the average MOE for ZEN fell below 10,000, suggesting a potential health risk. Furthermore, extended exposure over 20 years resulted in a substantial reduction in the average ZEN MOE to 259. Notably, chronic dietary consumption persisting beyond 50 years demonstrated a critical decrease in average MOE values below 100, indicating a significantly elevated risk level (Figure 1E,G).

Considering that Coix seed is used as a medicinal material by taking its decoction, we further investigated the ZEN content in the decoctions of 50 randomly selected batches of Coix seed. The results demonstrated that ZEN could be transferred from raw Coix seed samples to their corresponding decoctions, with an average transfer rate of 5.30% (Figure 1H–J). A risk assessment of ZEN in Coix seed decoctions was conducted. The results indicated that the estimated daily intake of ZEN increased progressively with prolonged exposure duration. Specifically, in the medicinal scenario, when the exposure time reached 20 years, the average EDI was 0.65 ng/kg bw (50th = 0.43 ng/kg bw, 95th = 0.92 ng/kg bw), and it increased with the extension of exposure time (Figure 1K,M). The MOE value exhibited a gradual decline with increasing exposure duration. When the exposure duration through medicinal intake exceeded 10 years, the average MOE for ZEN ranged between 100 and 10,000, suggesting potential health risks. For a 20-year exposure period, the average MOE for ZEN was 4880 (Figure 1L,N), indicating a higher level of potential health risk.

### 3.2. Risk Assessment for AFs in Coix Seed for Dietary Consumption and Medicinal Intake

**Figure 2:**
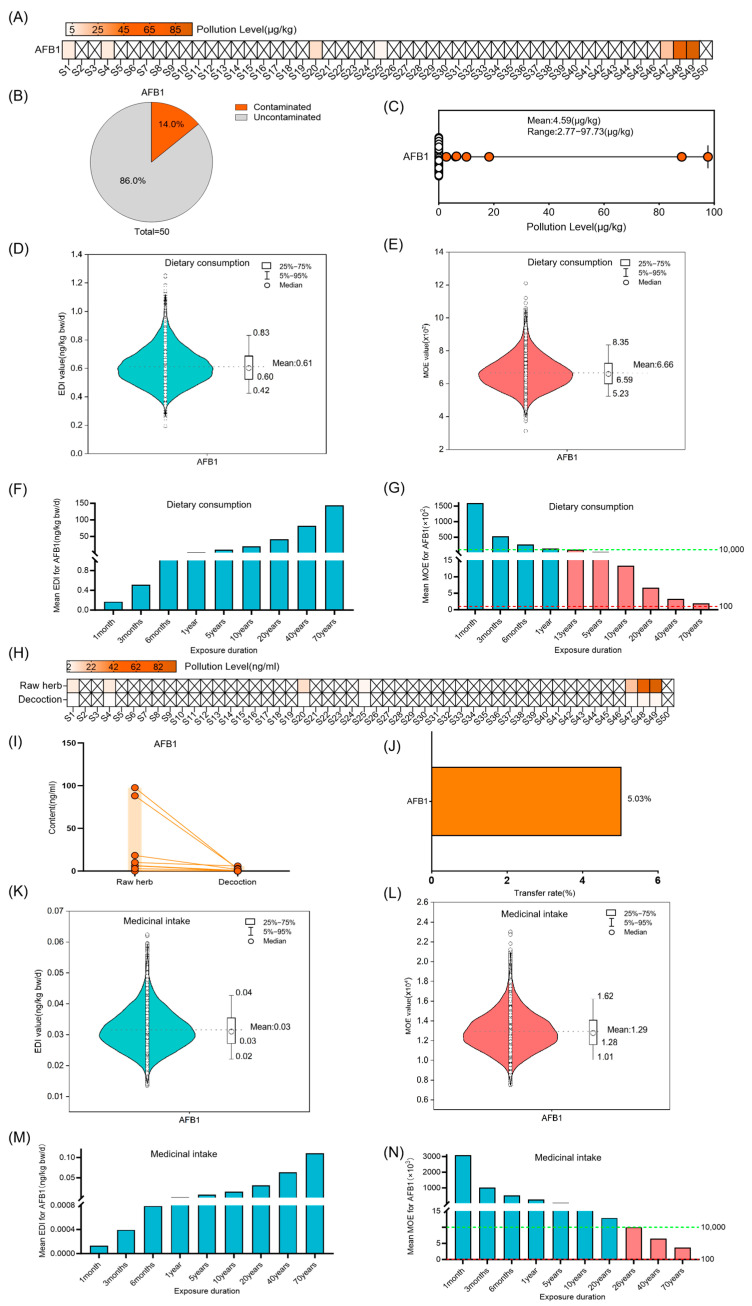
Risk assessment for AFB1 in Coix seed for dietary consumption and medicinal intake. (**A**–**C**) Concentration and occurrence of AFB1 in 50 random Coix seed samples. The symbol “×” denotes concentrations below the limit of quantification (LOQ). (**D**,**E**) The distribution of Estimated Daily Intake (EDI) and Margin of Exposure (MOE) of AFB1 in Coix seed via dietary exposure for 20 years (10,000 simulations). The violin shape represents the density distribution of EDI/MOE values. The dashed line marks the mean value as a reference. (**F**,**G**) The average EDI and MOE of AFB1 in Coix seed for dietary consumption over different exposure durations. The dashed green line marks the MOE reference value of 10,000, and the dashed red line marks the MOE reference value of 100. Different colors of bars represent distinct MOE ranges: teal bars indicate MOE > 10,000, pink bars indicate MOE between 100 and 10,000. (**H**–**J**) Quantification of AFB1 in 50 random Coix seed samples and their decoctions, and analysis of its transfer (concentration and rate) from the raw herb to the decoction. The symbol “×” denotes concentrations below the limit of quantification (LOQ). Each circle represents the mycotoxin detection value of an individual sample, and the red lines connect the paired samples from the same batch. (**K**,**L**) The distribution of Estimated Daily Intake (EDI) and Margin of Exposure (MOE) of AFB1 in decoction via medicinal exposure for 20 years (10,000 simulations). The violin shape represents the density distribution of EDI/MOE values. The dashed line marks the mean value as a reference. (**M**,**N**) The average EDI and MOE of AFB1 in the decoction of Coix seed for medicinal intake over different exposure durations. The dashed green line marks the MOE reference value of 10,000, and the dashed red line marks the MOE reference value of 100. Different colors of bars represent distinct MOE ranges: teal bars indicate MOE > 10,000, pink bars indicate MOE between 100 and 10,000.

AFB1 was present in 14.0% of the raw Coix seed samples, at levels ranging from 2.77 to 97.7 μg/kg and an average concentration of 4.58 ± 18.52 μg/kg (Figure 2A–C). The average EDI from Coix seed consumption was 0.61 ng/kg bw (50th = 0.60 ng/kg bw, 95th = 0.83 ng/kg bw) (Figure 2D,F). When the exposure duration exceeds 1 year, the average MOE value for AFB1 falls below 10,000, indicating potential health risks. With a 20-year exposure period, the average MOE value drops to 666, showing increased risk with longer exposure (Figure 2E,G). The transfer rate of AFB1 from raw Coix seed material to its decoction was determined to be 5.03% (Figure 2H–J). As the exposure duration extended, the EDI value gradually increased, whereas the MOE value progressively decreased. When medicinal exposure exceeded 40 years, the mean MOE for AFB1 ranged between 100 and 10,000, indicating a moderate risk level (Figure 2K–N).

**Figure 3:**
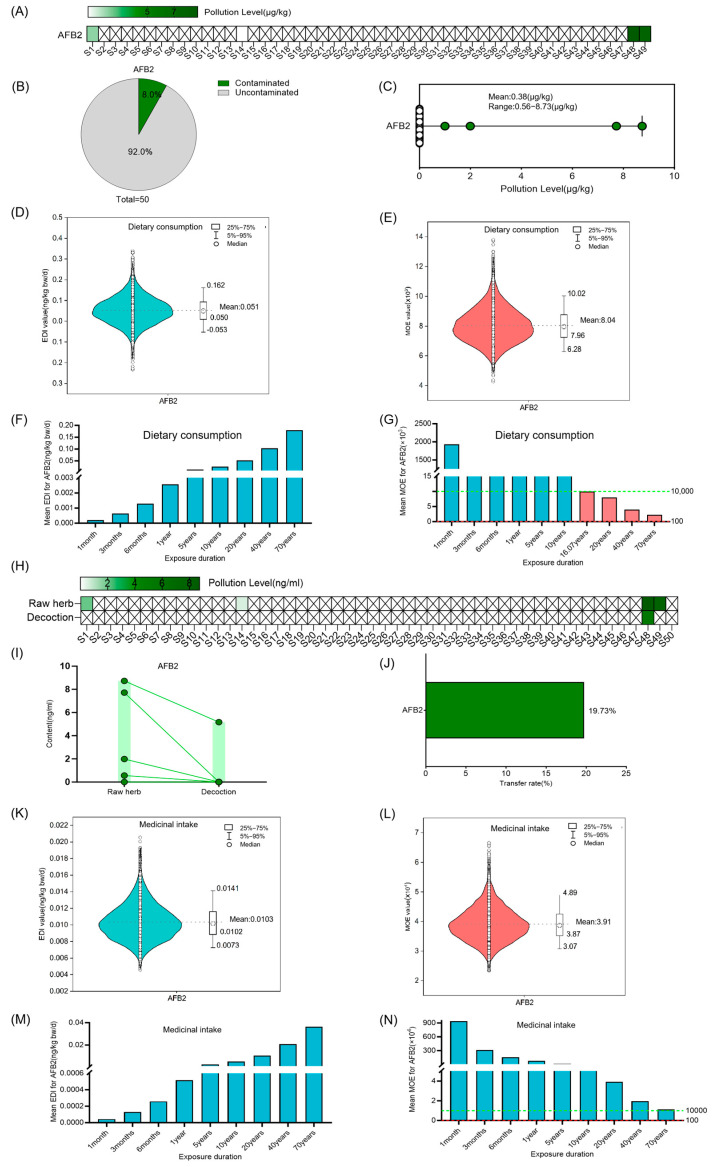
Risk assessment for AFB2 in Coix seed for dietary consumption and medicinal intake. (**A**–**C**) Concentration and occurrence of AFB2 in 50 random Coix seed samples. The symbol “×” denotes concentrations below the limit of quantification (LOQ). (**D**,**E**) The distribution of Estimated Daily Intake (EDI) and Margin of Exposure (MOE) of AFB2 in Coix seed via dietary exposure for 20 years (10,000 simulations). The violin shape represents the density distribution of EDI/MOE values. The dashed line marks the mean value as a reference. (**F**,**G**) The average EDI and MOE of AFB2 in Coix seed for dietary consumption over different exposure durations. The dashed green line marks the MOE reference value of 10,000, and the dashed red line marks the MOE reference value of 100. Different colors of bars represent distinct MOE ranges: teal bars indicate MOE > 10,000, pink bars indicate MOE between 100 and 10,000. (**H**–**J**) Quantification of AFB2 in 50 random Coix seed samples and their decoctions, and analysis of its transfer (concentration and rate) from the raw herb to the decoction. The symbol “×” denotes concentrations below the limit of quantification (LOQ). Each circle represents the mycotoxin detection value of an individual sample, and the lines connect the paired samples from the same batch. (**K**,**L**) The distribution of Estimated Daily Intake (EDI) and Margin of Exposure (MOE) of AFB2 in decoction via medicinal exposure for 20 years (10,000 simulations). The violin shape represents the density distribution of EDI/MOE values. The dashed line marks the mean value as a reference. (**M**,**N**) The average EDI and MOE of AFB2 in the decoction of Coix seed for medicinal intake over different exposure durations. The dashed green line marks the MOE reference value of 10,000, and the dashed red line marks the MOE reference value of 100.

AFB2 was present in 8.0% of the raw Coix seed samples, at levels ranging from 0.56 to 8.73 µg/kg and a mean concentration of 0.3 ± 1.6 µg/kg (Figure 3A–C). The average EDI from Coix seed consumption was 0.051 ng/kg bw (50th = −0.053 ng/kg bw, 95th = 0.162 ng/kg bw) (Figure 3D,F). When the exposure duration exceeds 20 years but remains below 70 years, the average MOE value for AFB2 falls within the range of 100 to 10,000, indicating a moderate level of potential health risk. With a 70-year exposure period, the average MOE value remains above 10,000, indicating no significant health risk (Figure 3E,G). The transfer rate of AFB2 from raw Coix seed material to its corresponding decoction was determined as 19.73% (Figure 3H–J). As the exposure duration extended, the EDI value gradually increased, while the MOE value progressively decreased. Within a 70-year exposure period under medicinal intake scenarios, the average MOE for AFB2 was greater than 10,000, suggesting an acceptable health risk (Figure 3L,N).

### 3.3. Risk Assessment for ST, DON OTA and T-2 in Coix Seed for Dietary Consumption and Medicinal Intake

**Figure 4:**
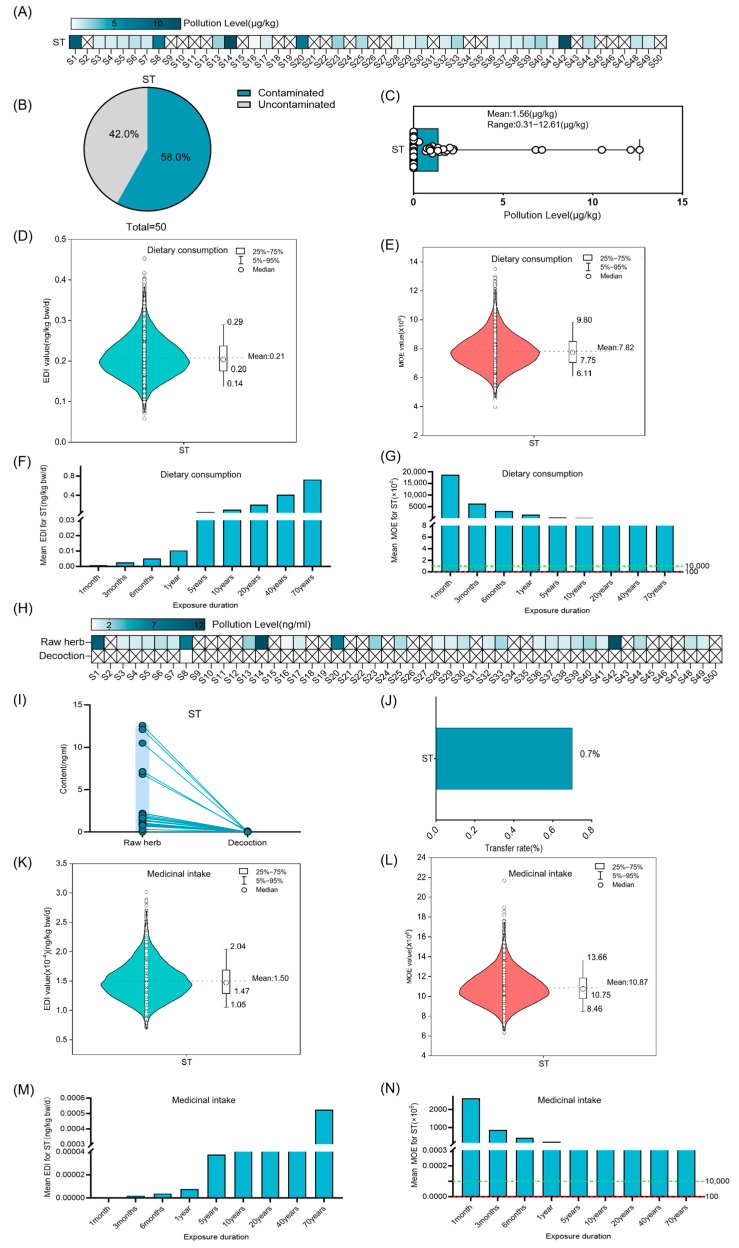
Risk assessment for ST in Coix seed for dietary consumption and medicinal intake. (**A**–**C**) Concentration and occurrence of ST in 50 random Coix seed samples. The symbol “×” denotes concentrations below the limit of quantification (LOQ). (**D**,**E**) The distribution of Estimated Daily Intake (EDI) and Margin of Exposure (MOE) of ST in Coix seed via dietary exposure for 20 years (10,000 simulations). The violin shape represents the density distribution of EDI/MOE values. The dashed line marks the mean value as a reference. (**F**,**G**) The average EDI and MOE of ST in Coix seed for dietary consumption over different exposure durations. The dashed green line marks the MOE reference value of 10,000, and the dashed red line marks the MOE reference value of 100. (**H**–**J**) Quantification of ST in 50 random Coix seed samples and their decoctions, and analysis of its transfer (concentration and rate) from the raw herb to the decoction. The symbol “×” denotes concentrations below the limit of quantification (LOQ). Each circle represents the mycotoxin detection value of an individual sample, and the lines connect the paired samples from the same batch. (**K**,**L**) The distribution of Estimated Daily Intake (EDI) and Margin of Exposure (MOE) of ST in decoction via medicinal exposure for 20 years (10,000 simulations). The violin shape represents the density distribution of EDI/MOE values. The dashed line marks the mean value as a reference. (**M**,**N**) The average EDI and MOE of ST in the decoction of Coix seed for medicinal intake over different exposure durations. The dashed green line marks the MOE reference value of 10,000, and the dashed red line marks the MOE reference value of 100.

The contamination rate of ST in Coix seed was 58.0%, with a mean contamination level of 1.56 ± 2.9 µg/kg (Figure 4A–C). Risk assessment revealed that the EDI of ST increased progressively with prolonged exposure duration. MOE values consistently exceeded 10,000 throughout a 70-year exposure period, suggesting no significant health risk (Figure 4D–G). The transfer rate of ST from raw Coix seed samples to corresponding decoctions was quantified as 0.7% (Figure 4H–J). Subsequent risk assessment demonstrated progressive elevation of EDI values with extended exposure duration in decoctions (Figure 4K,M), while MOE values remained above 10,000 through 70 years of medicinal exposure, indicating acceptable risk (Figure 4L,N).

**Figure 5:**
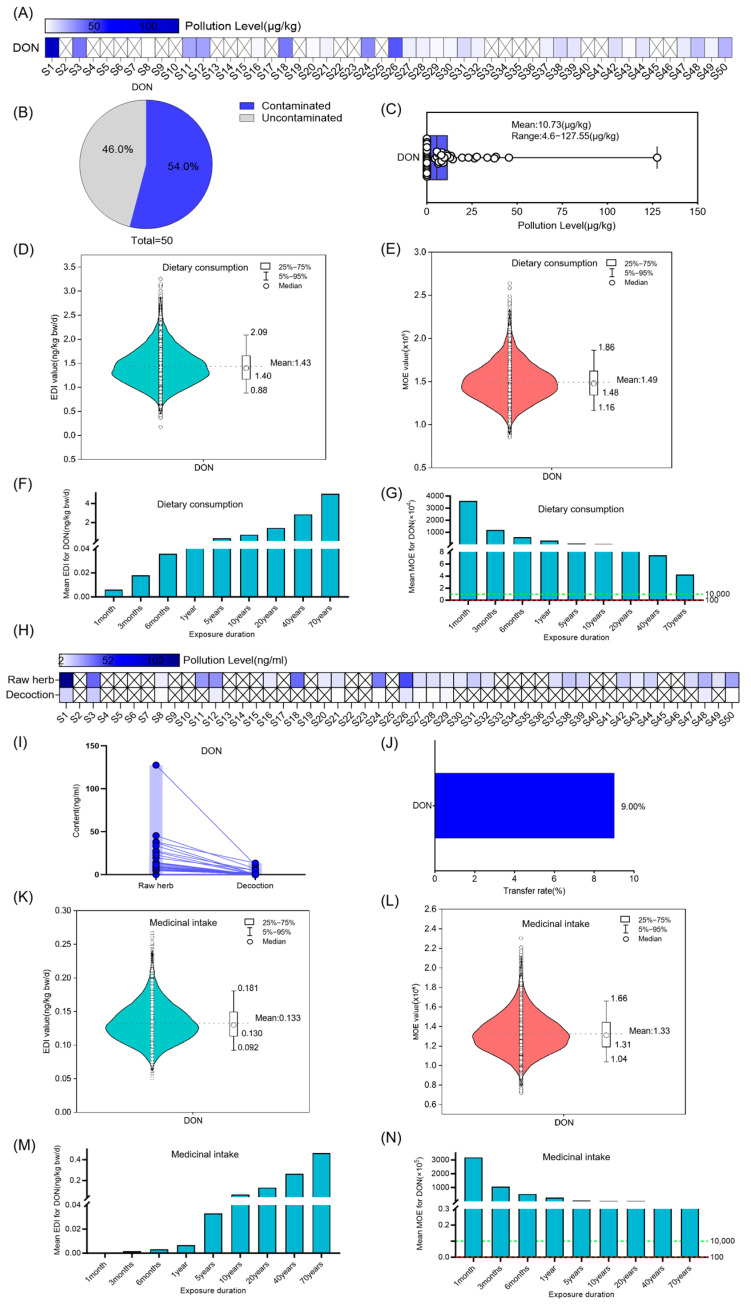
Risk assessment for DON in Coix seed for dietary consumption and medicinal intake. (**A**–**C**) Concentration and occurrence of DON in 50 random Coix seed samples. The symbol “×” denotes concentrations below the limit of quantification (LOQ). (**D**,**E**) The distribution of Estimated Daily Intake (EDI) and Margin of Exposure (MOE) of DON in Coix seed via dietary exposure for 20 years (10,000 simulations). The violin shape represents the density distribution of EDI/MOE values. The dashed line marks the mean value as a reference. (**F**,**G**) The average EDI and MOE of DON in Coix seed for dietary consumption over different exposure durations. The dashed green line marks the MOE reference value of 10,000, and the dashed red line marks the MOE reference value of 100. (**H**–**J**) Quantification of DON in 50 random Coix seed samples and their decoctions, and analysis of its transfer (concentration and rate) from the raw herb to the decoction. The symbol “×” denotes concentrations below the limit of quantification (LOQ). Each circle represents the mycotoxin detection value of an individual sample, and the lines connect the paired samples from the same batch. (**K**,**L**) The distribution of Estimated Daily Intake (EDI) and Margin of Exposure (MOE) of DON in decoction via medicinal exposure for 20 years (10,000 simulations). The violin shape represents the density distribution of EDI/MOE values. The dashed line marks the mean value as a reference. (**M**,**N**) The average EDI and MOE of DON in the decoction of Coix seed for medicinal intake over different exposure durations. The dashed green line marks the MOE reference value of 10,000, and the dashed red line marks the MOE reference value of 100.

For DON, a contamination incidence of 54.0% was observed in Coix seed, with mean concentrations measuring 10.73 ± 20.44 µg/kg (Figure 5A–C). The risk assessment profile showed gradual increases in DON EDI correlating with exposure duration, while MOE values maintained thresholds above 10,000 over seven decades, confirming acceptable risk levels (Figure 5D–G). Analytical determination revealed a 9.00% transfer rate of DON from raw materials to decoctions (Figure 5H–J). Decoction risk evaluation demonstrated time-dependent EDI escalation (Figure 5K,M), with sustained MOE values exceeding 10,000 through 70-year medicinal exposure scenarios (Figure 5L,N).

**Figure 6:**
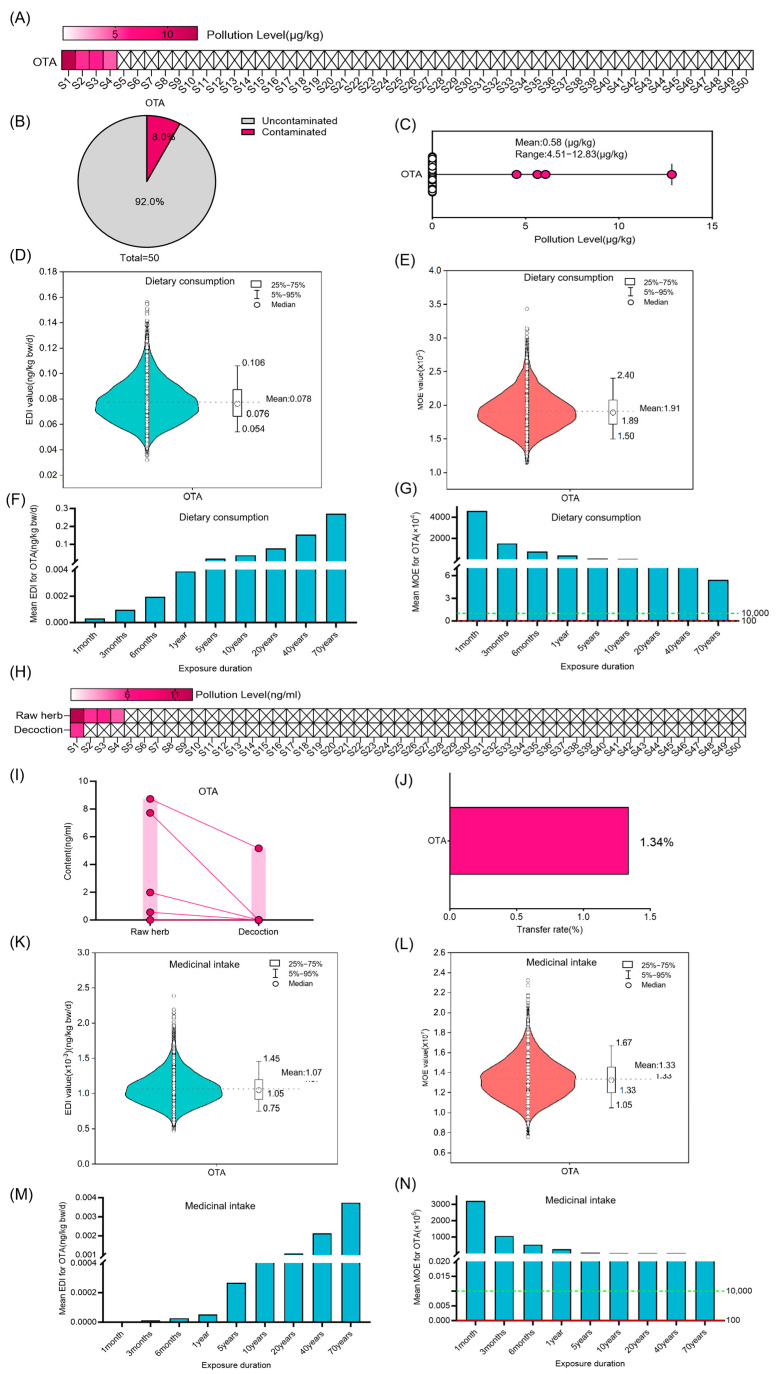
Risk assessment for OTA in Coix seed for dietary consumption and medicinal intake. (**A**–**C**) Concentration and occurrence of OTA in 50 random Coix seed samples. The symbol “×” denotes concentrations below the limit of quantification (LOQ). (**D**,**E**) The distribution of Estimated Daily Intake (EDI) and Margin of Exposure (MOE) of OTA (neoplastic effects) in Coix seed via dietary exposure for 20 years (10,000 simulations). The violin shape represents the density distribution of EDI/MOE values. The dashed line marks the mean value as a reference. (**F**,**G**) The average EDI and MOE of OTA in Coix seed for dietary consumption over different exposure durations. The dashed green line marks the MOE reference value of 10,000, and the dashed red line marks the MOE reference value of 100. (**H**–**J**) Quantification of DON in 50 random Coix seed samples and their decoctions, and analysis of its transfer (concentration and rate) from the raw herb to the decoction. The symbol “×” denotes concentrations below the limit of quantification (LOQ). Each circle represents the mycotoxin detection value of an individual sample, and the lines connect the paired samples from the same batch. (**K**,**L**) The distribution of Estimated Daily Intake (EDI) and Margin of Exposure (MOE) of OTA (neoplastic effects) in decoction via medicinal exposure for 20 years (10,000 simulations). The violin shape represents the density distribution of EDI/MOE values. The dashed line marks the mean value as a reference. (**M**,**N**) The average EDI and MOE of OTA in the decoction of Coix seed for medicinal consumption over different exposure durations. The dashed green line marks the MOE reference value of 10,000, and the dashed red line marks the MOE reference value of 100.

OTA was present in 8.0% of the Coix seed samples at a mean concentration of 0.58 ± 2.20 µg/kg (Figure 6A–C). The EDI of OTA exhibited gradual elevation with extended exposure duration, while MOE values persistently surpassed 10,000 during 70-year assessments, indicating negligible risk (Figure 6D–G). Decoction analysis identified a 1.34% transfer rate for OTA (Figure 6H–J). Subsequent risk evaluation confirmed progressive EDI increases with exposure duration (Figure 6K,M), with MOE values remaining above critical thresholds through seven decades of medicinal exposure (Figure 6L,N). Additional assessment of OTA’s non-neoplastic effects demonstrated MOE values substantially exceeding 10,000, even beyond 70-year exposure periods, indicating an acceptable risk (Figure S3A,B).

**Figure 7:**
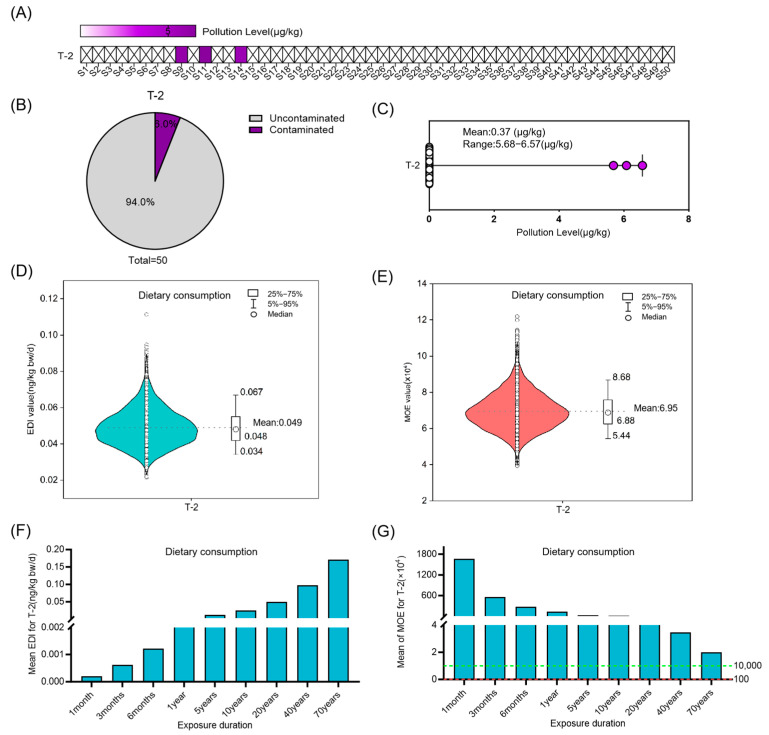
Risk assessment for T-2 in Coix seed for dietary consumption. (**A**–**C**) Concentration and occurrence of T-2 in 50 random Coix seed samples. The symbol “×” denotes concentrations below the limit of quantification (LOQ). (**D**,**E**) The distribution of Estimated Daily Intake (EDI) and Margin of Exposure (MOE) of T-2 in Coix seed via dietary exposure for 20 years (10,000 simulations). The violin shape represents the density distribution of EDI/MOE values. The dashed line marks the mean value as a reference. (**F**,**G**) The average EDI and MOE of T-2 in Coix seed for dietary consumption over different exposure durations. The dashed green line marks the MOE reference value of 10,000, and the dashed red line marks the MOE reference value of 100.

T-2 toxin contamination occurred in 6.0% of samples at a mean concentration of 0.3 ± 1.47 µg/kg (Figure 7A–C). Risk assessment revealed progressive EDI increases with exposure duration, reaching 0.049 ng/kg body weight (50th = 0.034 ng/kg bw; 95th = 0.067 ng/kg bw) after two decades of dietary exposure (Figure 7D,F). MOE values consistently exceeded 10,000 throughout 70-year evaluations, suggesting negligible health risks (Figure 7E,G). Notably, T-2 toxin was not detected in any of the decoction samples.

### 3.4. Risk Assessment of Multi-Mycotoxin Co-Exposure in Coix Seed for Dietary Consumption and Medicinal Intake

**Figure 8:**
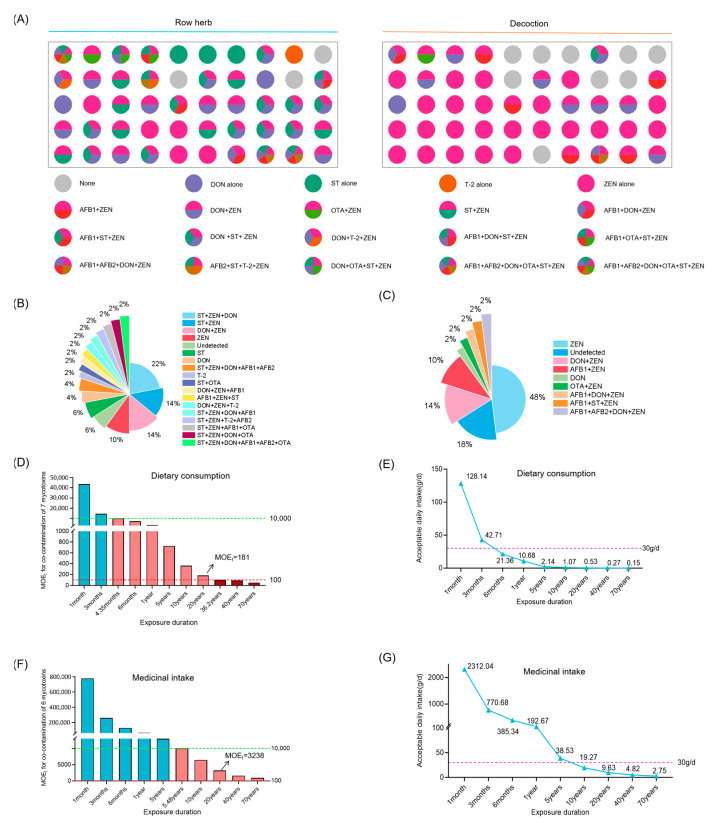
Risk assessment for mycotoxins co-exposure in Coix seed for dietary consumption and medicinal intake. (**A**–**C**) Concentration and occurrence of mycotoxins in 50 random samples, as well as the analysis of the combined characteristics of mycotoxin contamination in the raw herb and decoctions. (**D**,**E**) The average Margin of Exposure (MOE) and the acceptable daily intake (ADI) of various combinations of mycotoxin exposure in Coix seed for dietary consumption over different exposure durations. The dashed green line marks the MOE reference value of 10,000, and the dashed red line marks the MOE reference value of 100. Different colors of bars represent distinct MOE ranges: teal bars indicate MOE > 10,000, pink bars indicate MOE between 100 and 10,000, and red bars indicate MOE < 100. (**F**,**G**) The average Margin of Exposure (MOE) and the ADI of various combinations of mycotoxin exposure in Coix seed for medicinal intake over different exposure durations. The dashed green line marks the MOE reference value of 10,000, and the dashed red line marks the MOE reference value of 100. Different colors of bars represent distinct MOE ranges: teal bars indicate MOE > 10,000, pink bars indicate MOE between 100 and 10,000.

Among the 50 randomly selected samples of raw Coix seed, a total of seven mycotoxins were identified. Among these samples, 37 samples (74%) were found to be contaminated with two or more mycotoxins, and 56% of the contaminated samples exhibited co- exposure by toxins produced by both *Fusarium* and *Aspergillus* species. In the corresponding decoctions, 6 mycotoxins were identified, and 16 samples (32%) exhibited co- exposure (Figure 8A). Among the raw materials, the most common multi-toxin combination was “DON + ST + ZEN”, accounting for 21%, whereas in the decoctions, the predominant combination was “DON + ZEN”, representing 14% of samples (Figure 8B,C). Risk assessments for various toxin combinations were conducted using Equation (4), revealing that when all 7 mycotoxins were present together in raw materials, the average total margin of exposure (MOE_t_) was 181. Further analysis across different exposure durations indicated that when exposure exceeded 4.35 months (130 days), the MOE_t_ dropped below 10,000, suggesting a potential health risk. When exposure duration surpassed 40 years, the MOE_t_ fell below 100, indicating a significantly higher risk (Figure 8D). Additionally, Acceptable daily intake (ADI) was estimated. A continuous intake of 30 g/d for no more than 130 days is evaluated as the ADI for dietary consumption of Coix seed. If consumers continuously consumed Coix seed for 6 months (180 days), the ADI was reduced to 21.36 g (Figure 8E).

When Coix seed is used for medical purposes, analysis of different exposure durations indicates that the health risk is acceptable within 5.48 years (2000 days) of use (MOE > 10,000). Typically, Coix seed is administered medicinally for no more than 2–4 weeks, with long-term conditioning advised to be limited to within 3 months (Figure 8F). Therefore, under standard medicinal use scenarios, the health risks associated with mycotoxin exposure are considered acceptable. Calculations of ADI for continuous medicinal use indicated that at a usage duration of 3 months, the ADI was 770.68 g, which is substantially higher than the clinically recommended dosage of 30 g/day. This suggests that the risk associated with medicinal use within conventional treatment periods is acceptable. A continuous intake of 30 g/d for no more than 2000 days is evaluated as the ADI for medicinal use of Coix seed (Figure 8G). Furthermore, a dual-pathway risk assessment was performed based on the co-exposure level of mycotoxins in Coix seed using Equation (5), and the findings were similar with the aforementioned results (Figure S4A–D).

## 4. Discussion

The assessment shows that long-term dietary consumption of Coix seed may carry significant health risks, mainly due to ZEN, AFB1, AFB2, and the synergistic effects of multiple mycotoxin exposures. In contrast, only a small amount of mycotoxins transfer from the raw material to the aqueous decoction for medicinal intake of Coix seed. Given the typically short duration of medicinal use, associated health risks stay within acceptable limits. Additionally, intake thresholds for Coix seed are proposed to ensure negligible mycotoxin-related health risks.

Coix seed, with its rich nutritional profile, is highly susceptible to colonization by toxin-producing fungi. This fungal infestation further promotes the biosynthesis of mycotoxins, among which ZEN contamination is particularly widespread. Previous studies [17,18,35] reported a high incidence and levels of ZEN (59.6~97.5%) in regional Coix seed samples. Our investigation of 50 samples from five Chinese medicinal markets is consistent with these findings, quantifying ZEN in 82% of samples at a mean concentration of 298.11 µg/kg. The high contamination levels of ZEN in Coix seed can be primarily attributed to its nature as a gramineous crop, which is especially susceptible to *Fusarium* contamination [36]. The optimal growth conditions for *Fusarium* species capable of producing zearalenone (ZEN), such as *Fusarium* graminearum (25 to 30 °C, 0.90 water activity), overlap with the cultivation regions of Coix, resulting in a relatively high ZEN contamination level in Coix seed [37,38]. Other *Fusarium* mycotoxins found in our samples included DON (54%, 10.73 µg/kg) and T-2 (6%, 0.37 µg/kg); HT-2 was absent. This is consistent with the ecology of toxin-producing fungi, such as *F. sporotrichioides*, which favors temperatures of 3~7 °C, unlike the warmer climates where Coix is cultivated [39]. Notably, 52% of samples had ≥2 *Fusarium* mycotoxins, with “ZEN + DON” in 14%. In addition, Coix seed samples are also contaminated by *Aspergillus*, producing aflatoxins (AFs), OTA, and ST. Both literature data and our results indicate that sterigmatocystin (ST) has the highest incidence among *Aspergillus* toxins, with reported occurrence ranging from 29.9% to 82.5% in previous studies, compared to 58% quantified in our investigation [8,18], likely due to its producers’ adaptability to Coix growth conditions and limited conversion to aflatoxin precursors [32,40]. AFs and OTA, though less prevalent, are highly toxic and regulated globally [41]. *Aspergillus* co- exposure (≥2 toxins) was found in 18% of our samples, while 56% of samples had both *Fusarium* and *Aspergillus* mycotoxins (e.g., “ST + ZEN + DON” in 22%), highlighting widespread combined contamination. Such co-exposure may exacerbate toxic effects, complicating regulatory limit-setting. Therefore, a systematic risk assessment of both individual and co-exposure mycotoxin contamination is essential, as it serves as a scientific foundation for regulatory decision-making aimed at safeguarding public health.

Exposure to mycotoxins presents a direct or potential risk to human health, and extensive literature is available on their adverse effects in both human and animal populations [42,43]. Based on these findings, the seven mycotoxins identified in the Coix seed samples were evaluated in this study. The results indicate that long-term (20 years) consumption of Coix seed may pose health risks due to its contamination with ZEN or AFs (AFB1/AFB2) (MOE < 10,000). Particularly, when the exposure duration to ZEN exceeded 50 years, an MOE value below 100 was observed, indicating a high level of risk.

However, actual survey data revealed that 74% of Coix seed samples were simultaneously contaminated with two or more mycotoxins. Studies have demonstrated synergistic interactions among different mycotoxins [44,45]. A vitro experiments showed that combined exposure to DON and ZEN significantly reduced HepG2 cell viability and increased reactive oxygen species (ROS) levels, exhibiting a stronger toxic effect compared to exposure to either toxin alone [46]. A 3-week controlled diet study found no significant effects on body weight gain or average daily feed intake in piglets exposed to DON or ZEN individually; however, co-exposure resulted in a significant reduction in both parameters [47]. Similarly, combined exposure to AFB1 and ZEA caused greater adverse effects on immune function in goats and kidney cells in pigs than exposure to either toxin alone [48,49]. Therefore, in this study, we conducted the first risk assessment of mycotoxins co-exposure in Coix seed samples, considering both the characteristics of contaminant combinations and contamination levels. The health risks associated with ZEN and AFB1 in combination were 1.4 and 3.7 times higher, respectively, than those from exposure to ZEN or AFB1 alone, indicating an elevated risk under combined exposure conditions. Risk assessments across varying exposure durations revealed that exceeding a continuous consumption period of 4.35 months (130 days) led to surpassing the health risk threshold (MOE < 10,000). When the consumption duration extended beyond 35.57 years, the MOE dropped below 100, indicating a high-risk level. Considering variations in consumption duration and quantity among different consumer groups [50], this study innovatively proposed corresponding acceptable daily intake (ADI) tailored to specific consumption periods. The ADI for Coix seed consumption is established at 30 g/day for durations not exceeding 130 days. Notably, when dietary consumption periods are extended beyond 6 months, the ADI should fall below 21.36 g, representing a 28.8% reduction from the standard 30 g/day dosage [18]. These findings may serve as a reference for consumers to make informed decisions regarding appropriate intake levels and scientifically manage consumption duration when incorporating Coix seed into their diets.

Traditional Chinese medicine has been extensively utilized in healthcare systems across numerous countries and regions worldwide, with decoction serving as the most common preparation method for herbal formulations [51]. During the preparation of decoction in traditional Chinese medicine, the processes of decoction and filtration contribute to a reduction in mycotoxin content during transfer, thereby mitigating potential health risks. However, the transfer rates of mycotoxins vary among different Chinese medicinal materials. Yu et al. studied aflatoxin transfer in decoctions of five medicinal materials. AFB1 transfer rates were 4.37% (Lilii Bulbus), 8.79% (Bombyx Batryticatus), 14.19% (Nelumbinis Semen), 6.33% (Hordei Fructus Germinatus), 13.79% (Polygalae Radix)—Nelumbinis Semen was highest, Lilii Bulbus lowest. For AFB2, rates were 9.64% (Lilii Bulbus), 15.89% (Bombyx Batryticatus), 44.31% (Nelumbinis Semen), 29.77% (Hordei Fructus Germinatus), 22.92% (Polygalae Radix), with Nelumbinis Semen significantly higher [52]. Ying et al. reported a transfer rate of 56.1% for OTA in Astragalus Radix decoction [21], while another study found a transfer rate of 16.78% for OTA in Radix Dipsaci [25], showing OTA transfer varies by material. No existing literature covers mycotoxin transfer in Coix seed decoction, so this study is the first. Results showed 6 out of 7 mycotoxins in raw Coix seed transferred to the decoction: ZEN (5.30%), ST (0.70%), DON (9.00%), AFB1 (5.03%), AFB2 (19.73%), OTA (1.34%). These findings confirm mycotoxin transfer rates vary greatly among Chinese medicinal materials, possibly due to matrix components (e.g., polarity, compositional complexity) affecting mycotoxin dissolution and transfer.

Based on the transfer rate data, this study conducted a risk assessment of six mycotoxins in Coix seed under medicinal usage conditions. Results showed only ZEN posed a potential health risk (average MOE = 4880). Though boiling significantly reduced mycotoxin levels in Coix seed decoctions, co- exposure remained (32%). Thus, a comprehensive risk assessment of mycotoxin co-exposure was conducted, considering contamination profiles and concentrations. Findings showed the MOE value is 3238, indicating potential health risks with 20-year continuous use. Similarly to dietary exposure assessments, the health risk from co-exposure Coix seed via medicinal use was 1.5 times higher than that from ZEN alone. Unlike dietary consumption, medicinal Coix seed use has defined dosage and treatment duration. At the recommended daily dose of 30 g, the MOE value far exceeded 10,000 within the standard treatment period (3 months) [29,53], suggesting acceptable health risk. Considering that special patient groups may require extended treatment cycles or increased dosages [54], this study further evaluated risks across different administration durations. The findings indicate that potential health risks may emerge following continuous consumption of Coix seed decoctions exceeding 5.48 years (2000 days). Based on the recommended daily dose, an acceptable daily intake (ADI) of 30 g/day was established for medicinal applications of Coix seed, with a maximum recommended exposure duration of 2000 days. Given that medicinal use rarely exceeds this duration, the health risks associated with Coix seed in medicinal applications are deemed acceptable.

## 5. Conclusions

This study systematically evaluated the contamination status of multiple mycotoxins in Coix seeds and the associated health risks via dietary and medicinal exposure pathways. The results indicate that Coix seeds are highly susceptible to contamination by mycotoxins produced by fungi of the genera *Fusarium* and *Aspergillus*. Among the eight targeted mycotoxins, ZEN was the most prevalent contaminant. Co-exposure to multiple mycotoxins was common, with two or more detected in 74% of samples. Risk assessment indicates that long-term consumption (>20 years) may pose potential health risks, primarily due to individual or combined exposure to ZEN, AFB1 and AFB2. The risk associated with co-exposure to multiple mycotoxins is higher than that of single-mycotoxin contamination. In medicinal applications, mycotoxin transfer rates during the decoction process were relatively low, and health risks remained within acceptable limits under standard dosages (30 g/day) and treatment durations (≤3 months). This study established, for the first time, a comprehensive mycotoxin risk assessment model for Coix seeds in both food and medicinal contexts, proposing ADI values based on varying exposure durations. This research establishes reference parameters for Coix seed dosage and application duration as a dual-use substance; its co-occurring toxin profiles inform methodological development for composite exposure risk evaluation.

## Data Availability

The original contributions presented in the study are included in the article, further inquiries can be directed to the corresponding authors.

## Supplementary Materials

The following supporting information can be downloaded at: https://www.mdpi.com/article/10.3390/foods14223965/s1, Figure S1: Optimization of the QuEChERS Pretreatment Method for Coix Seed. Figure S2. Specific chromatogram of 8 mycotoxins, counts per second. Figure S3. Contamination levels and risk assessment of OTA in Coix seed raw herb and their decoctions. Figure S4. Dual-pathway risk assessment based on the contamination characteristics of co-exposure to mycotoxins in Coix seed. Table S1. Mass Spectrometry Parameters for 8 Mycotoxins. Table S2. Calibration and Validation of the Analytical Method for Simultaneous Detection of 8 Mycotoxins in Raw Coix Seed. Table S3. Calibration and Validation of the Analytical Method for Simultaneous Detection of 8 Mycotoxins in Coix Seed Decoction. Table S4. Repeatability, Stability, and Precision of the Determination of 8 Mycotoxins in Raw Coix Seed and Coix Seed Decoction. Table S5. Recovery of 8 kinds of mycotoxins from Raw Coix Seed and Coix Seed Decoction (*n* = 3). Table S6. The BMDL10/NOAEL Values for 8 Mycotoxins.

## Abbreviations

The following abbreviations are used in this manuscript:

ADI	Acceptable daily intake
AFB1	Aflatoxin B1
AFB2	Aflatoxin B2
Afs	Aflatoxins
AY	Average Life Expectancy (Year)
BMDL_10_	Benchmark Dose Lower Confidence Limit (for a 10% response)
BW	Average Body Weight
DON	Deoxynivalenol
ED	Exposure Duration
EDI	Estimated Daily Intake
HT-2	HT-2 Toxin
IC	Daily intake of Coix seed
LOD	Limit of Detection
LOQ	Limit of Quantification
MD	Mycotoxin Content in Decoction
MOE	Margin of Exposure
MR	Mycotoxin Content in Raw Material
MRM	Multiple Reaction Monitoring
NOAEL	No-Observed-Adverse-Effect Level
OTA	Ochratoxin A
ST	Sterigmatocystin
T-2	T-2 Toxin
ZEN	Zearalenone
