# Supplementary material for: Mycotoxins Co-Exposure Risk Assessment in Coix Seed: Contamination Levels and Safety for Dietary Consumption and Medicinal Intake
Source: Foods. 2025 Nov 19;14(22):3965. doi: 10.3390/foods14223965 (PMC12651520; doi:10.3390/foods14223965)
Supplement: Supplementary file 1 [file foods-14-03965-s001.zip › foods-3956777-supplementary.pdf]

Supplementary figures and tables

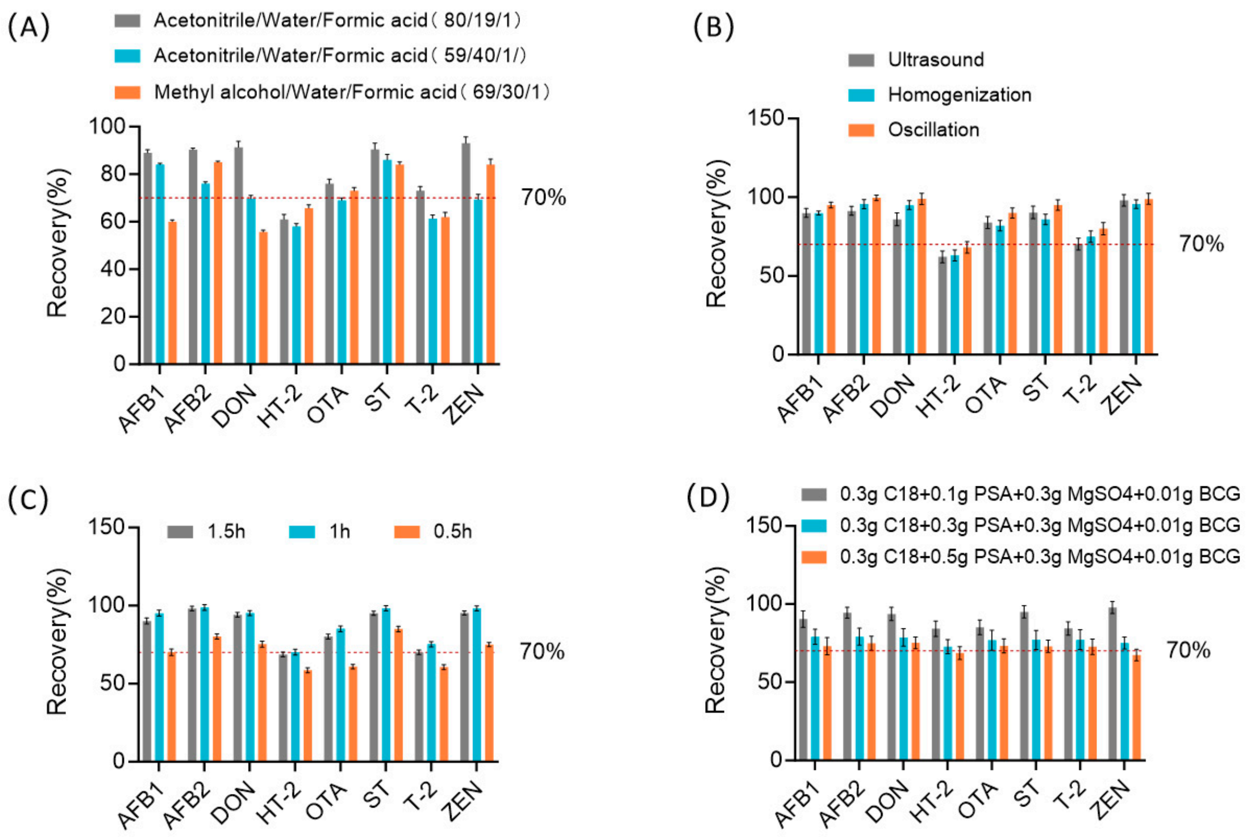

Figure S1. Optimization of the QuEChERS Pretreatment Method for Coix Seed.(A) extraction solvent,(B) extraction method, (C) Oscillation extraction time and (D)purifying agent. Recovery .

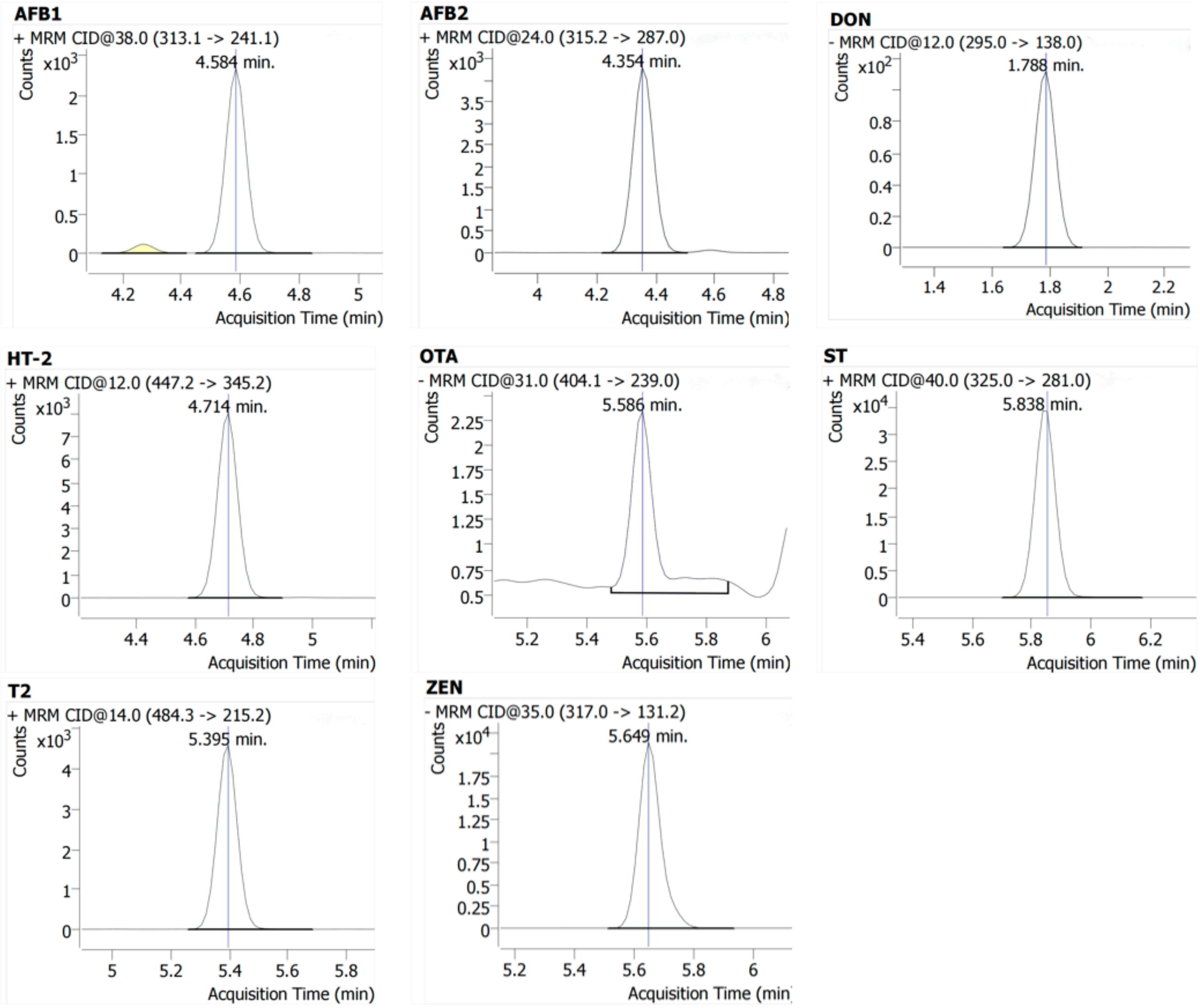

Figure S2. Specific chromatogram of 8 mycotoxins , counts per second.

Table S1. Mass Spectrometry Parameters for 8 Mycotoxins

Reference substance	Parent ion *m/z*	Daughter ion *m/z*	Tapered bore voltage /eV	Collision Voltage /eV	Acquisition Mode
AFB1	313.1	285.0[1)] /241.0[2)]	30	25/30	$[M+H]^+$
AFB2	315.1	287.0[1)] /259.0[2)]	30	25/20	$[M+H]^+$
OTA	404.1	238.9[1)] /340.9[2)]	20	15/25	$[M+H]^+$
ZEN	319.1	283.0[1)] /301.0[2)]	10	15/10	$[M+H]^+$
DON	297.1	175.0[1)] /161.0[2)]	8	20/20	$[M+H]^+$
HT-2	447.0	285.0[1)] /345.0[2)]	25	22/20	$[M+Na]^+$
T-2	489.0	245.0[1)] /327.0[2)]	22	28/22	$[M+Na]^+$
ST	325.0	309.9[1)] /253.9[2)]	30	20/30	$[M+H]^+$

Table S2. Calibration and Validation of the Analytical Method for Simultaneous Detection of 8 Mycotoxins in Raw Coix Seed.

Mycotoxin	*Calibration curve	R^2	Linear range(ng)	LOD ($\mu g\cdot kg^{-1}$)	LOQ ($\mu g\cdot kg^{-1}$)	SEE(%)
AFB1	Y=12423.21X-1016.23	0.997 8	0.049~25	0.013	0.027	90.02
AFB2	Y=22134.44X-1255.22	0.999 2	0.039~10	0.020	0.051	105.59
DON	Y=1761.32X-492.51	0.998 9	3.906~500	0.611	1.354	67.78
HT-2	Y=3552.69X-1012.86	0.994 9	1.953~500	0.411	1.054	117.68
OTA	Y=8423.33X-621.12	0.998 9	0.039~10	0.010	0.021	110.22
ST	Y=14125.34X-876.19	0.999 4	0.936~100	0.089	0.106	107.47
T-2	Y=2225.11X+158.89	0.996 3	0.195~100	0.046	0.106	113.11
ZEN	Y=5890.15X-1782.41	0.999 1	0.098~50	0.026	0.053	120.87

*Calibration curves were adjusted for matrix effects. LOD,limit of detection; LOQ,limit of quantitation;SSE,suppression/enhancement values.

Table S3. Calibration and Validation of the Analytical Method for Simultaneous Detection of 8 Mycotoxins in Coix Seed Decoction.

Mycotoxin	*Calibration curve	R^2	Linear range(ng)	LOD ($\mu g\cdot kg^{-1}$)	LOQ ($\mu g\cdot kg^{-1}$)	SEE(%)
AFB1	Y=13264X+461.51	0.998 6	0.049~25	0.012	0.026	96.32
AFB2	Y=13423.03X-467.18	0.999 4	0.039~10	0.010	0.030	102.18
DON	Y=1659.25X-132.404	0.997 4	15.624~500	1.163	2.532	73.52
HT-2	Y=6739.07X-913.07	0.999 4	3.906~500	0.393	1.006	105.91
OTA	Y=6732.76X-1056.14	0.999 6	0.039~10	0.009	0.020	117.94
ST	Y=3593.15X-901.62	0.999 2	0.195~100	0.084	0.101	115.89

T-2	Y=3479.13X+1306.44	0.999 0	0.195~100	0.044	0.101	121.12
ZEN	Y=3057.95X-3592.03	0.997 7	0.39~50	0.025	0.050	127.13

*Calibration curves were adjusted for matrix effects. LOD,limit of detection; LOQ,limit of quantitation;SSE,suppression/enhancement values.

Table S4. Repeatability, Stability, and Precision of the Determination of 8 Mycotoxins in Raw Coix Seed and Coix Seed Decoction.

Mycotoxin	Raw Coix Seed			Coix Seed Decoction		
	Reproducibility (%)	Stability (%)	Precision (%)	Reproducibility (%)	Stability (%)	Precision (%)
AFB1	5.49	5.36	1.25	0.25	4.38	2.35
AFB1	4.66	2.67	5.93	4.33	4.72	1.56
DON	1.26	3.09	2.59	3.64	1.04	3.31
HT-2	2.47	0.70	0.53	1.01	1.12	2.35
OTA	0.79	0.31	5.65	0.86	1.34	3.63
ST	3.67	2.78	3.73	3.49	4.66	1.16
T-2	3.45	4.40	3.73	0.44	3.49	1.56
ZEN	4.11	1.71	3.09	1.96	1.89	2.04

Table S5. Recovery of 8 kinds of mycotoxins from Raw Coix Seed and Coix Seed Decoction（n=3）.

Mycotoxin	Raw Coix Seed				Coix Seed Decoction			
	Low (%)	Medium (%)	High (%)	*RSD (%)	Low (%)	Medium (%)	High (%)	RSD (%)
AFB1	73.14	85.49	88.41	2.8	99.2	96.4	99.5	0.95
AFB2	101.60	104.70	94.64	2.5	87.5	104.1	99	3.12
DON	103.50	116.20	102.80	3.6	87.3	98.7	98.2	2.28
HT-2	115.90	98.72	98.30	2.4	108.6	98.7	98	1.21
OTA	82.36	77.10	70.88	3.5	96.5	96.6	87.7	2.36
ST	101.18	97.61	112.23	2.6	102.6	96.6	100.7	1.75
T-2	104.30	103.50	113.10	1.9	113.4	94	101.1	2.56

ZEN	94.57	94.11	84.30	1.5	103.3	110.7	97.3	1.96

*RSD,relative standard deviation.

Table S6. The $BMDL_{10}$/NOAEL Values for 8 Mycotoxins

**Mycotoxins**	**$BMDL_{10}$/ NOAEL**
Aflatoxin B1	0.4 μg/kg bw/d
Aflatoxin B2	0.4 μg/kg bw/d
Deoxynivalenol	210 μg/kg bw/d
Ochratoxin A fornon - neoplastic effects	4.73 μg/kg bw/d
Ochratoxin A forneoplastic effects	14.5 μg/kg bw/d
Sterigmatocystin	160μg/kg bw/d
T-2 Toxin	3.33 μg/kg bw/d
Zearalenone	10.4 μg/kg bw/d （NOAEL）

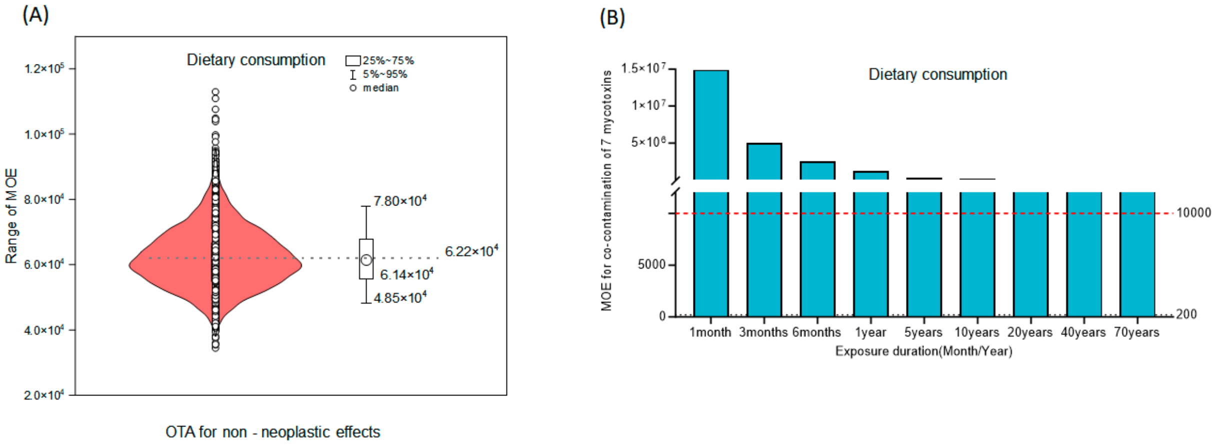

Figure S3. Contamination levels and risk assessment of OTA in Coix seed raw herb and their decoctions. **(A)**Violin plot and box plot of the distribution of MOE of OTA (neoplastic effects)

in Coix seed via dietary exposure for 20 years (10,000 simulations). **(B)** Bar chart of average MOE of OTA (neoplastic effects) in Coix seed raw herb for dietary consumption over different exposure durations.

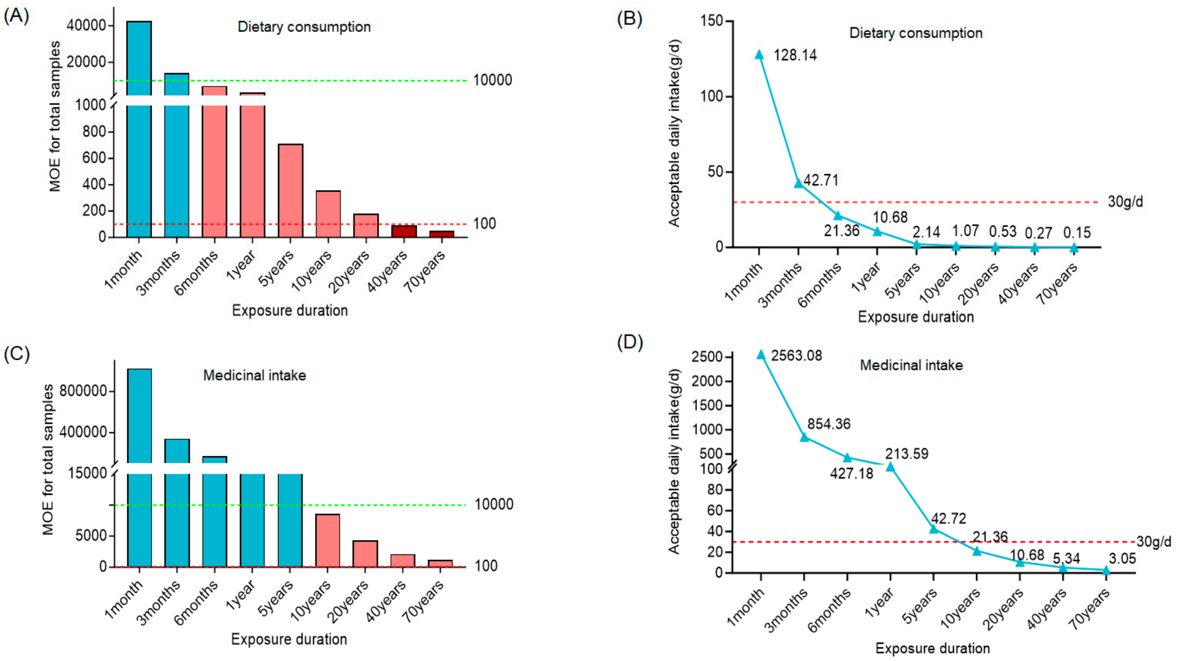

Figure S4. Dual-pathway risk assessment based on the contamination characteristics of co-exposure to mycotoxins in Coix seed.**(A-B)** The average Margin of Exposure (MOE) and the acceptable daily intake (ADI) of various combinations of mycotoxin exposure in Coix seed for dietary consumption over different exposure durations. **(C-D)** The average Margin of Exposure (MOE) and the ADI of various combinations of mycotoxin exposure in Coix seed for medicinal consumption over different exposure durations.
